# Supplementary material for: Vaccine Hesitancy among European Parents—Psychological and Social Factors Influencing the Decision to Vaccinate against HPV: A Systematic Review and Meta-Analysis
Source: Vaccines (Basel). 2024 Jan 26;12(2):127. doi: 10.3390/vaccines12020127 (PMC10891840; doi:10.3390/vaccines12020127)
Supplement: Supplementary file 1 [file vaccines-12-00127-s001.zip › Supplementary Material S2.pdf]

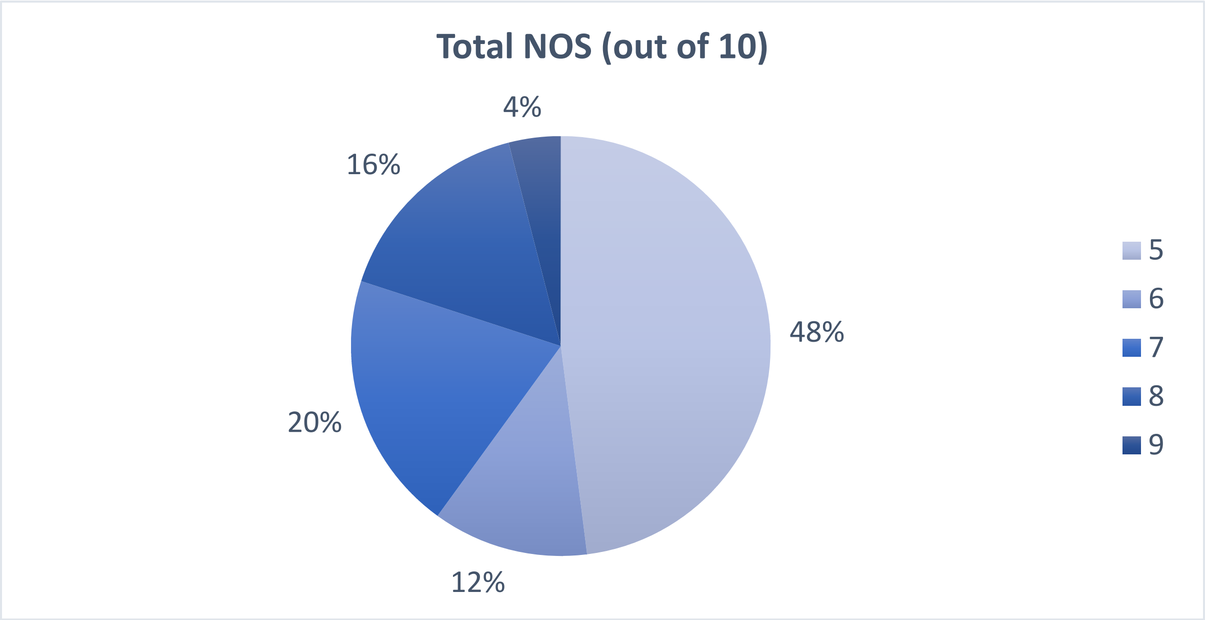

Chart 1: NOS scoring for the included publciations

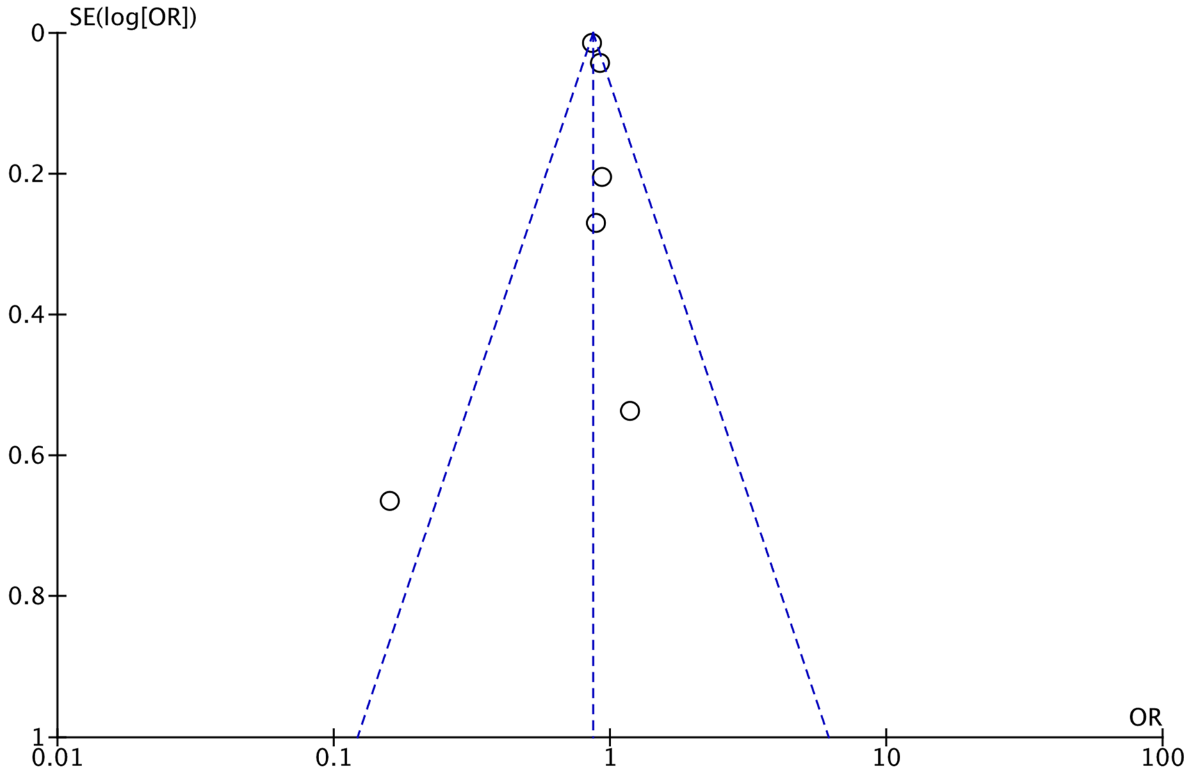

Figure S1: Funnel plot for the age of parent comparison

Table S1: Sensitivity analysis for the age of parent comparison

Study	Overall OR	Z	P	95% CI	I2	Initial CI
Brabin et al 2006	0.86	9.56	< 0.00001	0.84 - 0.89	55%	0.84 - 0.89
Dahlstrom et al 2009	0.85	9.47	< 0.00001	0.83 - 0.88	43%	
Donders et al 2007	0.86	9.58	< 0.00001	0.84 - 0.89	53%	
Haesebaert et al 2014	0.86	9.48	< 0.00001	0.84 - 0.89	0%	
Hansen et al 2015	0.91	2.26	0.02	0.83 - 0.99	44%	
Pelucchi et al 2010	0.86	9.57	< 0.00001	0.84 - 0.89	54%	

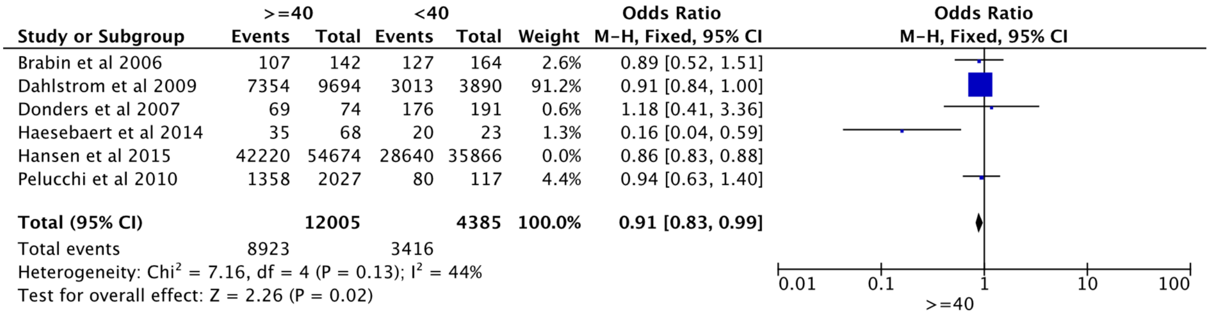

Figure S2: Forrest plot for the age of parent comparison excluding the Hansen et al. study

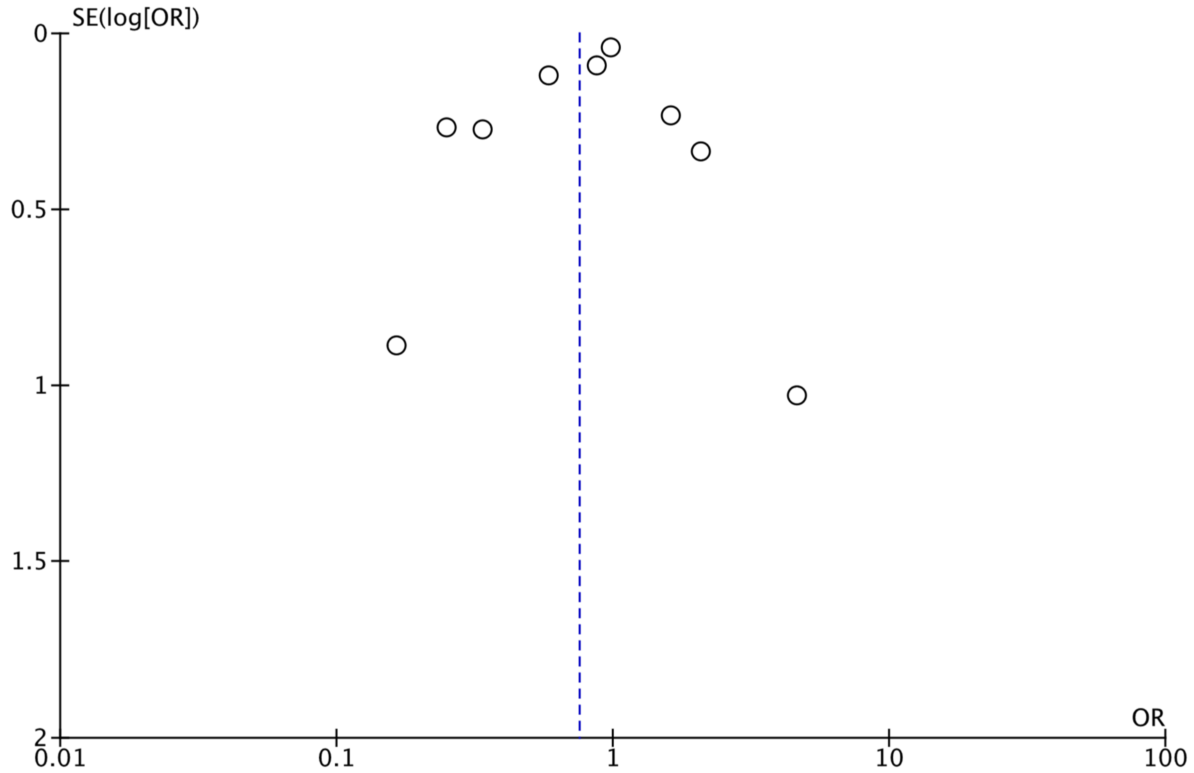

Figure S3: Funnel plot for the gender of parent comparison

Table S2: Sensitivity analysis for the gender of parent comparison

Study	Overall OR	Z	P	95% CI	I2	Initial CI
Dahlstrom et al 2009	0.72	1.40	0.16	0.45 - 1.14	88%	0.54 - 1.04
Ganczak et al 2018	0.67	2.30	0.02	0.48 - 0.94	89%	
Grandahl et al 2016	0.79	1.44	0.15	0.57 - 1.09	90%	
Lenselink et al 2007	0.72	1.95	0.05	0.52 - 1.00	90%	
Mari et al 2022	0.84	1.08	0.28	0.60 - 1.16	88%	
Pelucchi et al 2010	0.73	1.35	0.18	0.47 - 1.15	90%	
Sobierajski et al 2023	0.67	2.23	0.03	0.48 - 0.95	89%	
Sypien & Zielonka 2022	0.87	0.92	0.36	0.65 - 1.17	85%	
Waller et al 2019	0.79	1.28	0.20	0.55 - 1.13	88%	

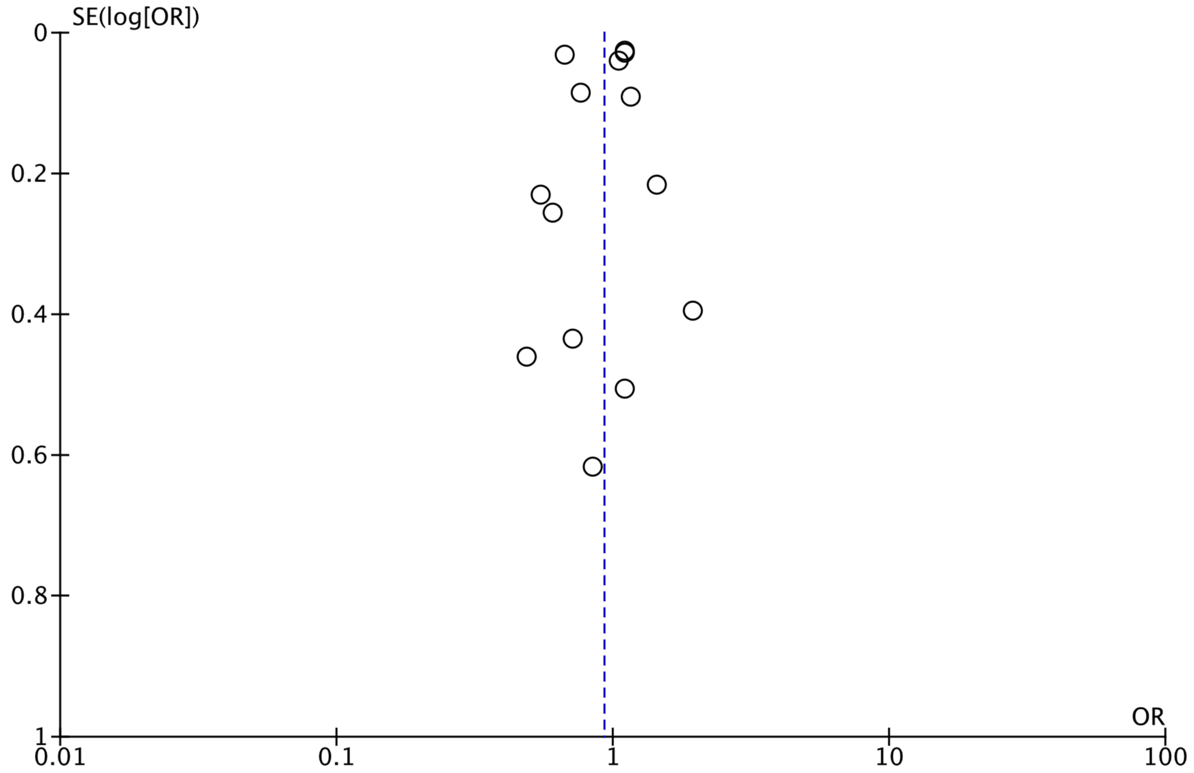

Figure S4: Funnel plot for the parent's educational level comparison

Table S3: Sensitivity analysis for the parent's educational level comparison

Study	Overall OR	Z	P	95% CI	I2	Initial CI
Baumann et al 2019	0.95	0.63	0.53	0.80 - 1.12	93%	0.79 - 1.09
Dahlstrom et al 2009	0.91	1.02	0.31	0.75 - 1.09	94%	
David et al 2022	0.92	0.98	0.33	0.78 - 1.08	94%	
Donders et al 2007	0.94	0.73	0.46	0.80 - 1.11	94%	
Feiring et al 2015	0.90	1.07	0.28	0.75 - 1.09	93%	
Gefenaite et al 2012	0.90	1.28	0.20	0.76 - 1.06	94%	
Grandahl et al 2016	0.93	0.92	0.36	0.79 - 1.09	94%	
Haesebaert et al 2014	0.90	1.23	0.22	0.77 - 1.06	94%	
Hansen et al 2015	0.90	1.09	0.28	0.75 - 1.09	92%	
Mari et al 2022	0.95	0.62	0.54	0.81 - 1.12	94%	
Pelucchi et al 2010	0.90	1.19	0.23	0.76 - 1.07	94%	
Rousset-Jablonski et al 2021	0.93	0.85	0.40	0.79 - 1.10	94%	
Schreiber et al 2015	1.01	0.14	0.89	0.91 - 1.11	70%	
Sobierajski et al 2023	0.96	0.51	0.61	0.81 - 1.13	94%	

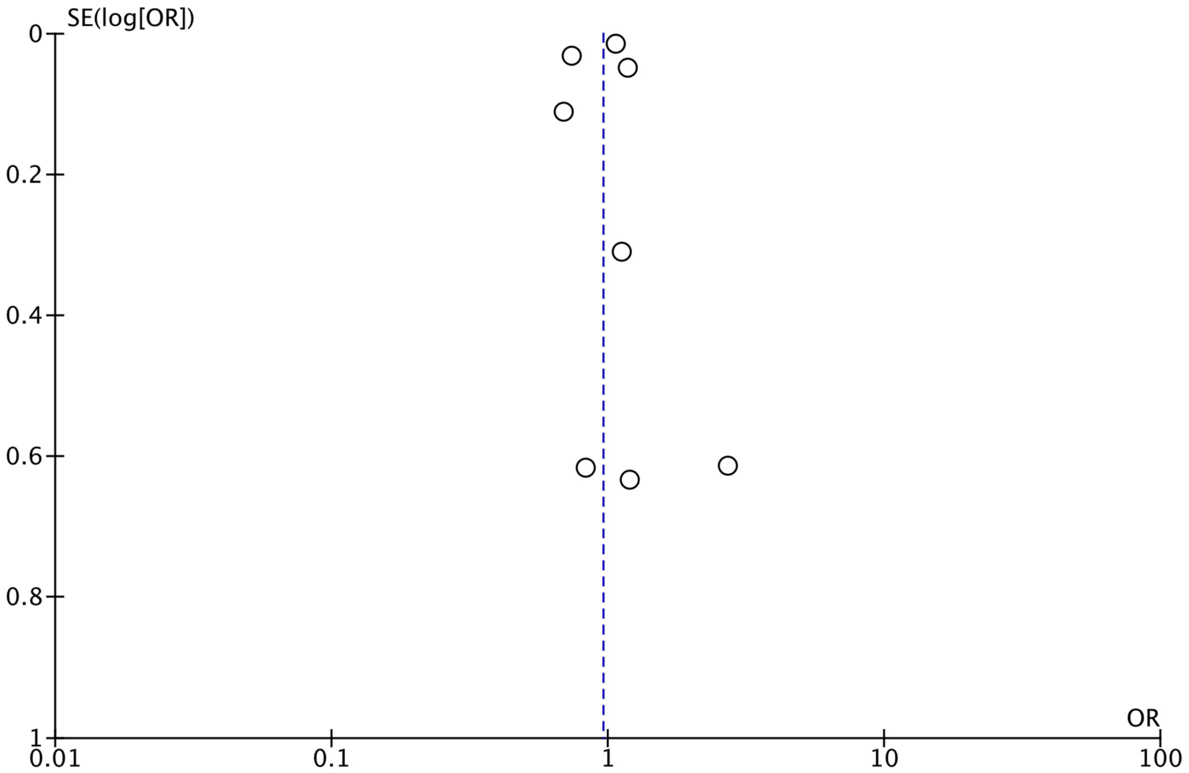

Figure S5: Funnel plot for the parent's civil status comparison

Table S4: Sensitivity analysis for parent's civil status comparison

Study	Overall OR	Z	P	95% CI	I2	Initial CI
Baumann et al 2019	1.03	0.22	0.82	0.81 - 1.30	95%	0.77 - 1.18
Dahlstrom et al 2009	0.91	0.71	0.48	0.70 - 1.18	95%	
Ganczak et al 2018	0.94	0.51	0.61	0.75 - 1.18	95%	
Grandahl et al 2016	0.96	0.36	0.72	0.77 - 1.19	95%	
Haesebaert et al 2014	0.93	0.67	0.50	0.75 - 1.15	95%	
Hansen et al 2015	0.95	0.32	0.75	0.70 - 1.29	92%	
Lenselink et al 2007	0.95	0.45	0.65	0.77 - 1.18	95%	
Schreiber et al 2015	1.02	0.29	0.78	0.87 - 1.20	74%	

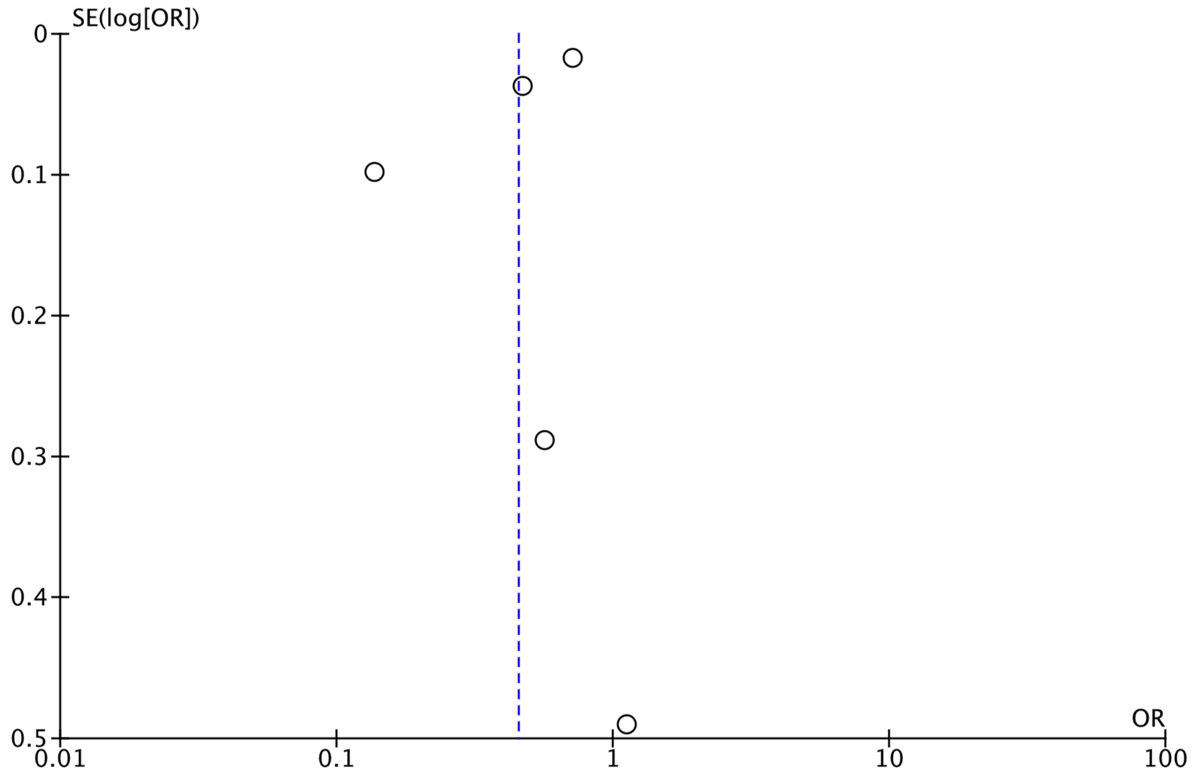

Figure S6: Funnel plot for parent employment status comparison

Table S5: Sensitivity analysis for parent employment status comparison

Study	Overall OR	Z	P	95% CI	I2	Initial CI
Baumann et al 2019	0.62	2.72	0.006	0.44 - 0.87	97%	0.28 - 0.74
Ganczak et al 2018	0.43	3.03	0.002	0.25 - 0.74	99%	
Haesebaert et al 2014	0.40	3.45	0.0006	0.24 - 0.67	99%	
Hansen et al 2015	0.42	2.01	0.04	0.18 - 0.98	98%	
Schreiber et al 2015	0.48	1.30	0.19	0.16 - 1.45	99%	

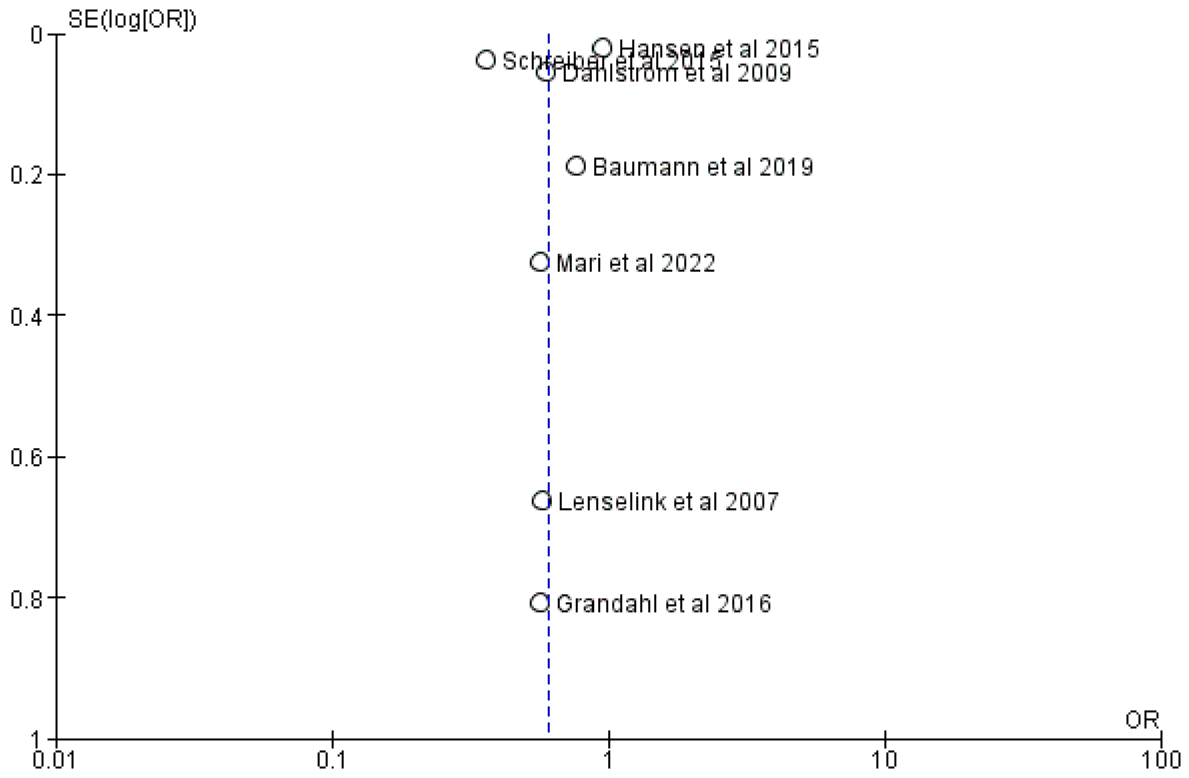

Figure S7: Funnel plot for the immigrant status comparison

Table S6: Sensitivity analysis for the immigrant status comparison

Study	Overall OR	Z	P	95% CI	I2	Initial CI
Baumann et al 2019	0.58	2.02	0.04	0.35 - 0.98	99%	0.38 - 0.98
Dahlstrom et al 2009	0.61	1.60	0.11	0.34 - 1.12	99%	
Grandahl et al 2016	0.61	1.96	0.05	0.38 - 1.00	99%	
Hansen et al 2015	0.54	3.40	0.0007	0.38 - 0.77	92%	
Lenselink et al 2007	0.61	1.95	0.05	0.38 - 1.00	99%	
Mari et al 2022	0.62	1.85	0.06	0.37 - 1.03	99%	
Schreiber et al 2015	0.71	2.07	0.04	0.51 - 0.98	92%	

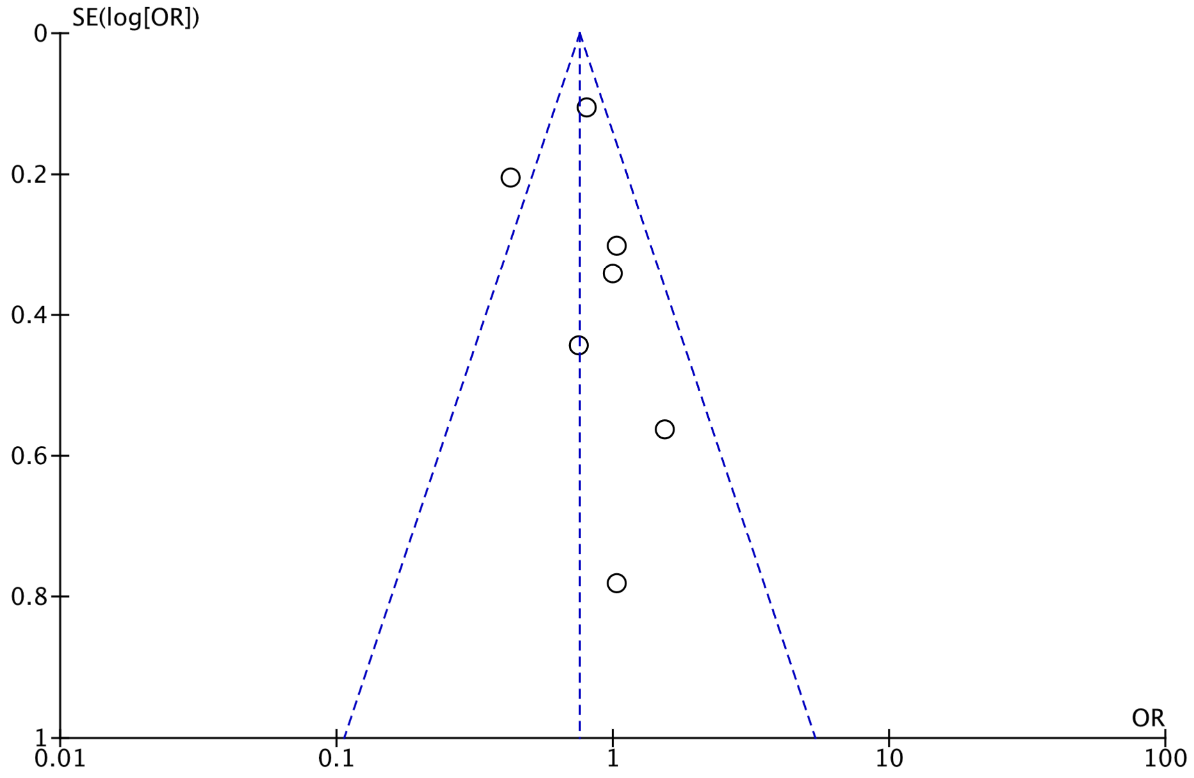

Figure S8: Funnel plot for the religious status of the responder's comparison

Table S7: Sensitivity analysis for the religious status of the responder's comparison

Study	Overall OR	Z	P	95% CI	I2	Initial CI
Bedford et al 2021	0.68	2.80	0.005	0.52 - 0.89	53%	0.64 - 0.89
Brabin et al 2006	0.74	3.50	0.0005	0.63 - 0.88	53%	
Ganczak et al 2018	0.75	3.42	0.0006	0.64 - 0.89	56%	
Gefenaite et al 2012	0.85	1.82	0.07	0.71 - 1.01	0%	
Grandahl et al 2016	0.74	3.54	0.0004	0.63 - 0.88	49%	
Lenselink et al 2007	0.75	3.33	0.0009	0.64 - 0.89	56%	
Sypien & Zielonka 2022	0.74	3.55	0.0004	0.62 - 0.87	52%	

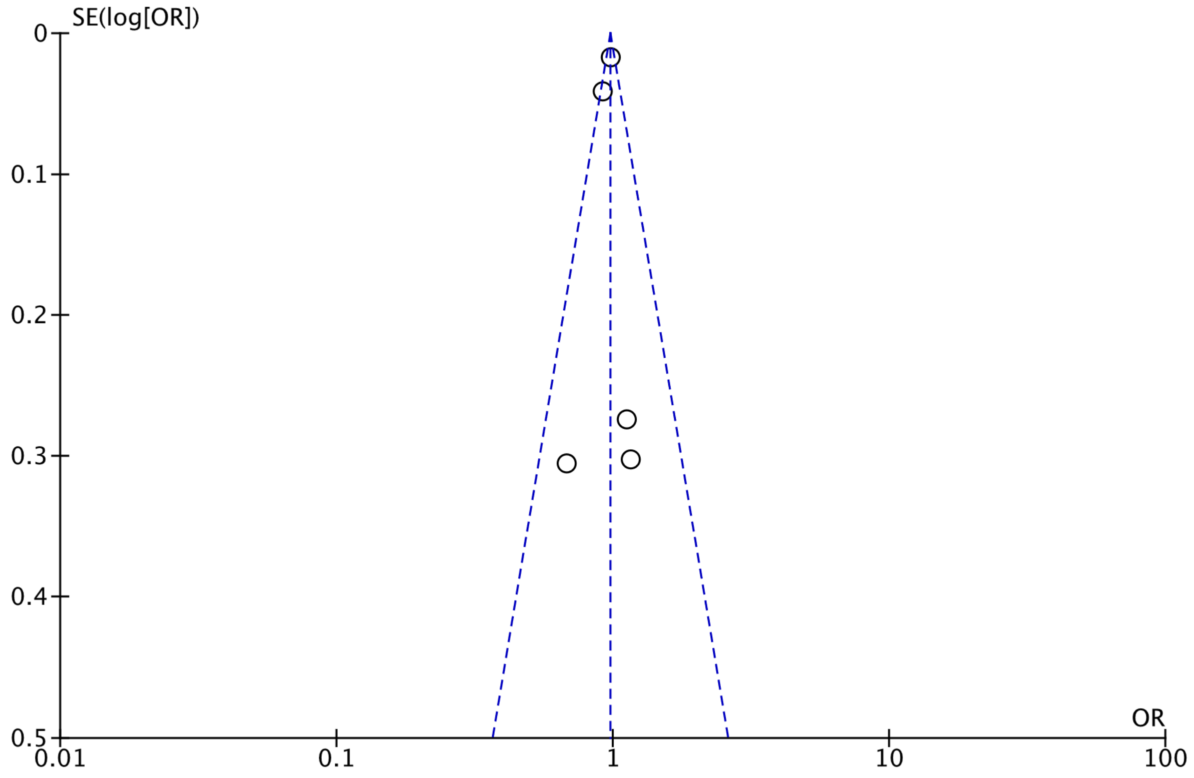

Figure S9: Funnel plot for the number of children comparison

Table S8: Sensitivity analysis for the number of children comparison

Study	Overall OR	Z	P	95% CI	I2	Initial CI
Brabin et al 2006	0.97	1.80	0.07	0.94 - 1.00	9%	
Dahlstrom et al 2009	0.98	1.18	0.24	0.95 - 1.01	0%	0.94 - 1.00
Ganczak et al 2018	0.97	1.80	0.07	0.94 - 1.00	8%	
Hansen et al 2015	0.93	1.80	0.07	0.86 - 1.01	0%	
Sypien & Zielonka 2022	0.97	1.70	0.09	0.94 - 1.00	0%	

Study or Subgroup	>2 children Events	>2 children Total	<=2 children Events	<=2 children Total	Weight	Odds Ratio M-H, Fixed, 95% CI
Brabin et al 2006	149	193	87	116	2.0%	1.13 [0.66, 1.93]
Dahlstrom et al 2009	3583	4751	6784	8833	94.2%	0.93 [0.85, 1.01]
Ganczak et al 2018	108	125	275	325	1.7%	1.16 [0.64, 2.09]
Hansen et al 2015	24300	31094	45886	58463	0.0%	0.98 [0.95, 1.01]
Sypien & Zielonka 2022	22	54	125	248	2.1%	0.68 [0.37, 1.23]
**Total (95% CI)**		**5123**		**9522**	**100.0%**	**0.93 [0.86, 1.01]**
Total events	3862		7271			

Heterogeneity: $Chi^2 = 2.11$, df = 3 (P = 0.55); $I^2 = 0\%$
Test for overall effect: Z = 1.80 (P = 0.07)

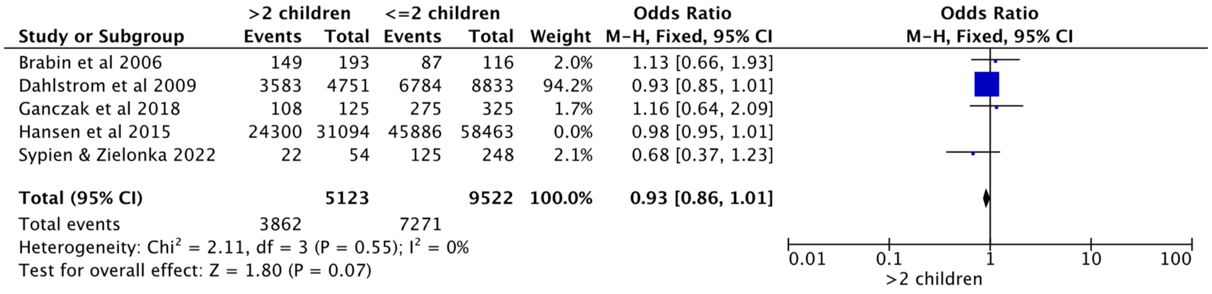

Figure S10: Forrest plot for the number of children comparison excluding the Hansen et al. study

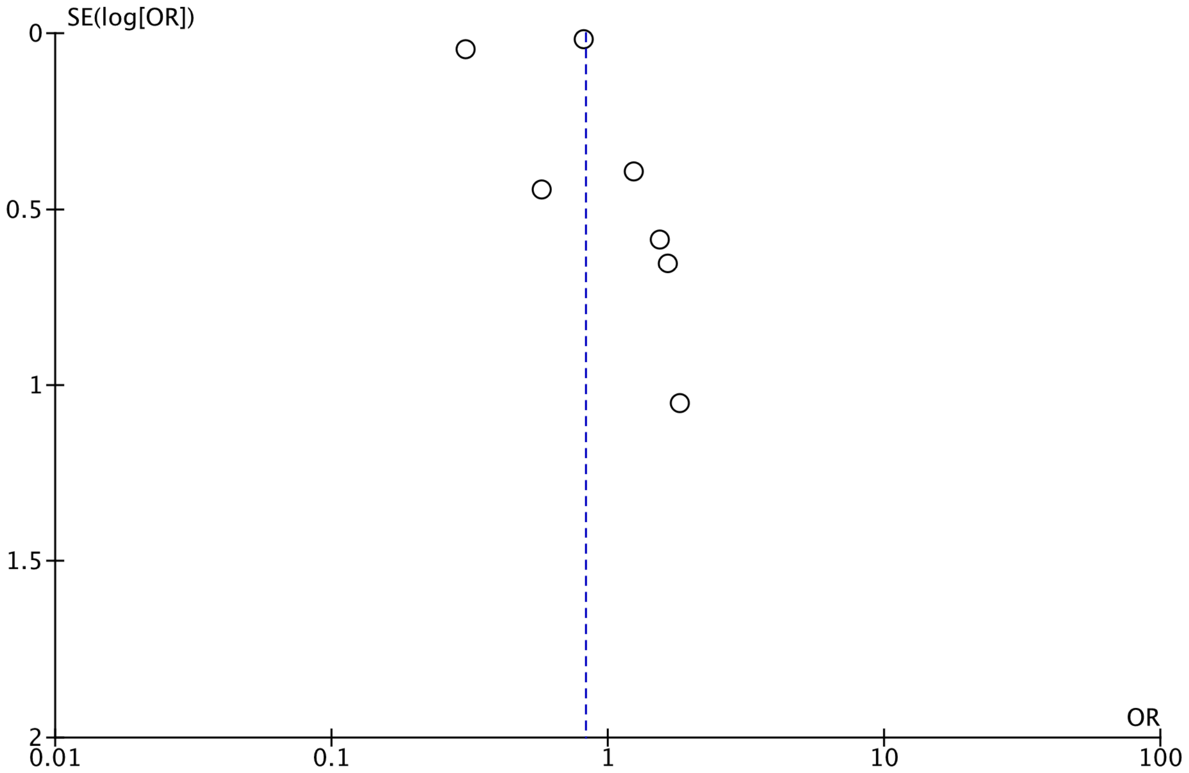

Figure S11: Funnel plot for the mother's history of CCS

Table S9: Sensitivity analysis for the mother's history of CCS

Study	Overall OR	Z	P	95% CI	I2	Initial CI
David et al 2022	0.76	0.83	0.41	0.40 - 1.45	99%	0.45 - 1.52
Gefenaite et al 2012	0.77	0.77	0.44	0.39 - 1.50	99%	
Haesebaert et al 2014	0.76	0.82	0.41	0.40 - 1.46	99%	
Hansen et al 2015	0.87	0.33	0.74	0.39 - 1.97	84%	
Lenselink et al 2007	0.79	0.75	0.45	0.42 - 1.48	99%	
Rousset-Jablonski et al 2021	0.88	0.37	0.71	0.45 - 1.71	99%	
Spencer et al 2007	0.82	10.20	< 0.00001	0.79 - 0.85	0%	

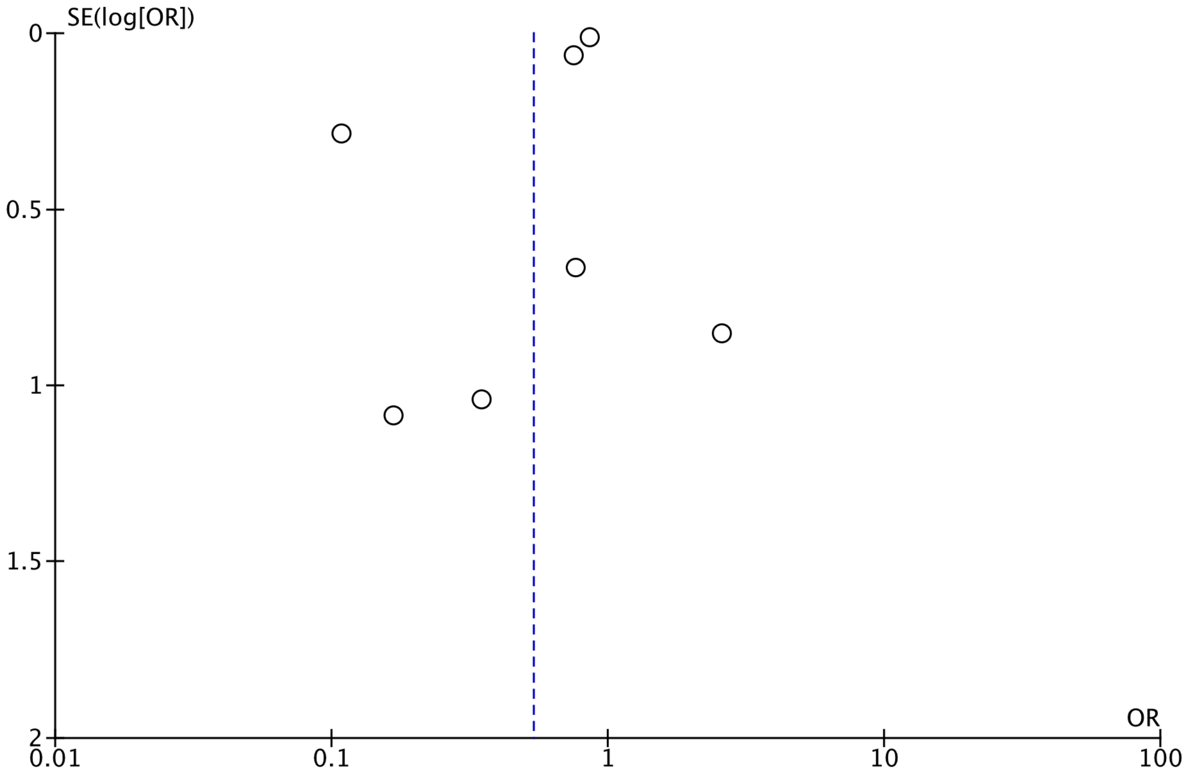

Figure S12: Funnel plot for the mother's Pap history comparison

Table S10: Sensitivity analysis for the mother's Pap history comparison

Study	Overall OR	Z	P	95% CI	I2	Initial CI
David et al 2022	0.50	3.47	0.0005	0.34 - 0.74	91%	0.36 - 0.79
Dib et al 2022	0.81	2.87	0.004	0.70 - 0.94	43%	
Haesebaert et al 2014	0.56	2.95	0.003	0.38 - 0.82	91%	
Hansen et al 2015	0.47	1.33	0.18	0.15 - 1.44	91%	
Lenselink et al 2007	0.54	3.03	0.002	0.37 - 0.81	92%	
Rousset-Jablonski et al 2021	0.52	3.16	0.002	0.35 - 0.78	92%	
Spencer et al 2007	0.45	1.46	0.14	0.16 - 1.31	89%	

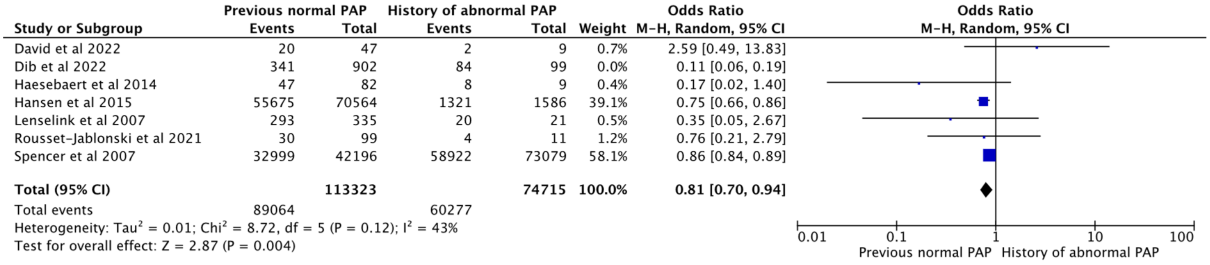

Figure S13: Forrest plot for the mother's Pap history comparison excluding the Dib et al. study

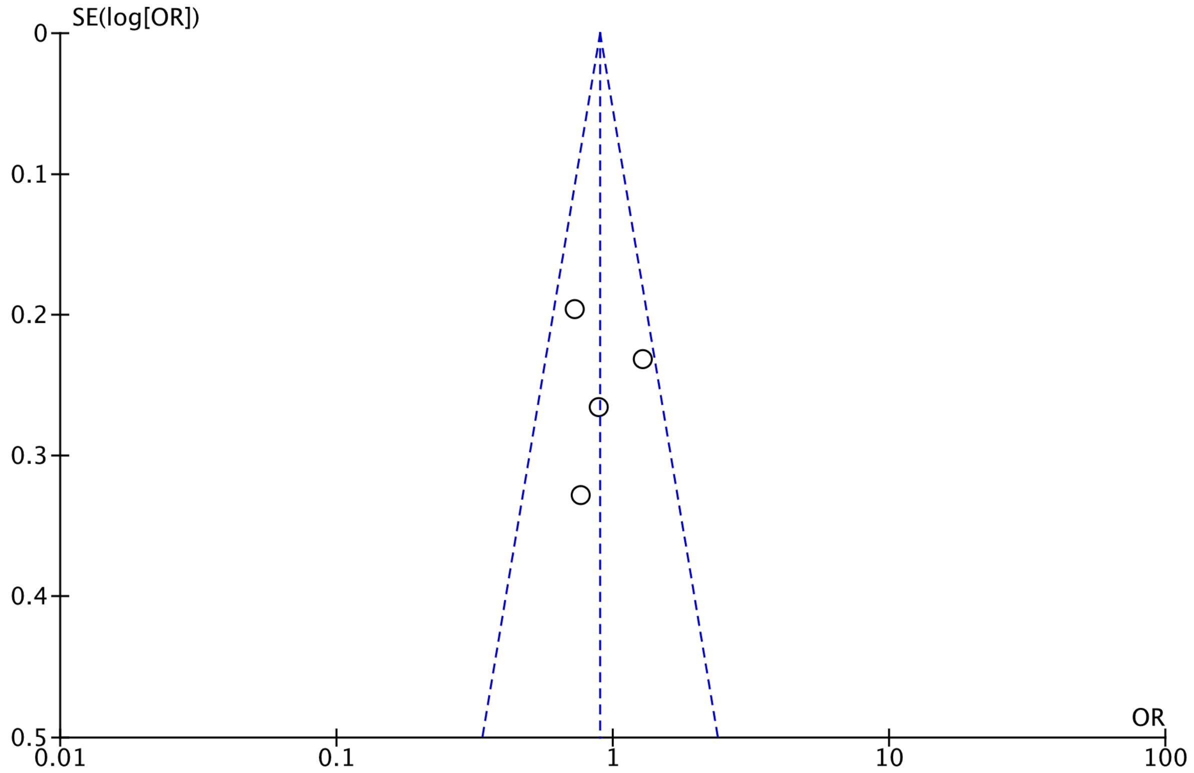

Figure S14: Funnel plot for the family history of cancer comparison

Table S11: Sensitivity analysis for the family history of cancer comparison

Study	Overall OR	Z	P	95% CI	I2	Initial CI
Ganczak et al 2018	0.89	0.83	0.41	0.68 - 1.17	48%	0.70 - 1.13
Gefenaite et al 2012	1.01	0.09	0.93	0.75 - 1.37	2%	
Lenselink et al 2007	0.91	0.69	0.49	0.71 - 1.18	44%	
Sypien & Zielonka 2022	0.78	1.78	0.08	0.59 - 1.03	0%	

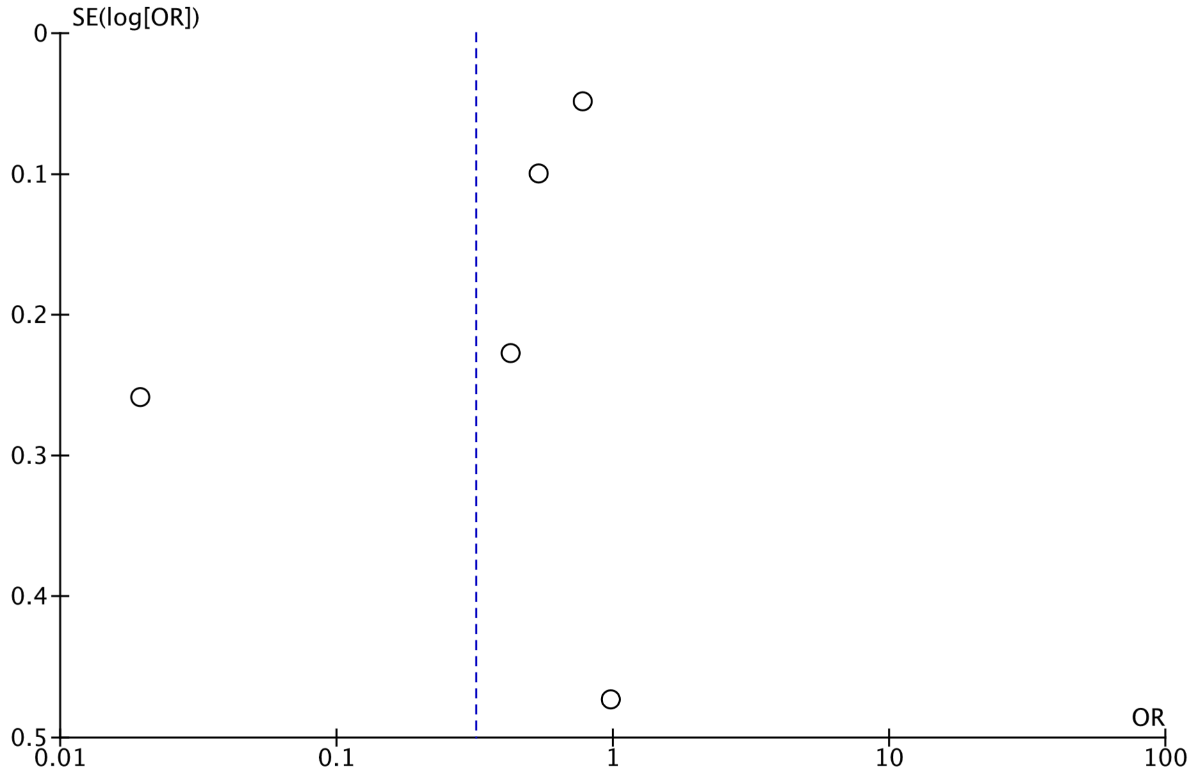

Figure S15: Funnel plot for the child's gender comparison

Table S12: Sensitivity analysis for the child's gender comparison

Study	Overall OR	Z	P	95% CI	I2	Initial CI
Dahlstrom et al 2009	0.25	1.75	0.08	0.05 - 1.18	98%	0.13 - 0.76
Donders et al 2007	0.62	3.01	0.003	0.45 - 0.85	82%	
Lenselink et al 2007	0.25	2.80	0.005	0.10 - 0.66	99%	
Pelucchi et al 2010	0.28	1.51	0.13	0.05 - 1.46	99%	
Sobierajski et al 2023	0.30	2.32	0.02	0.11 - 0.83	99%	

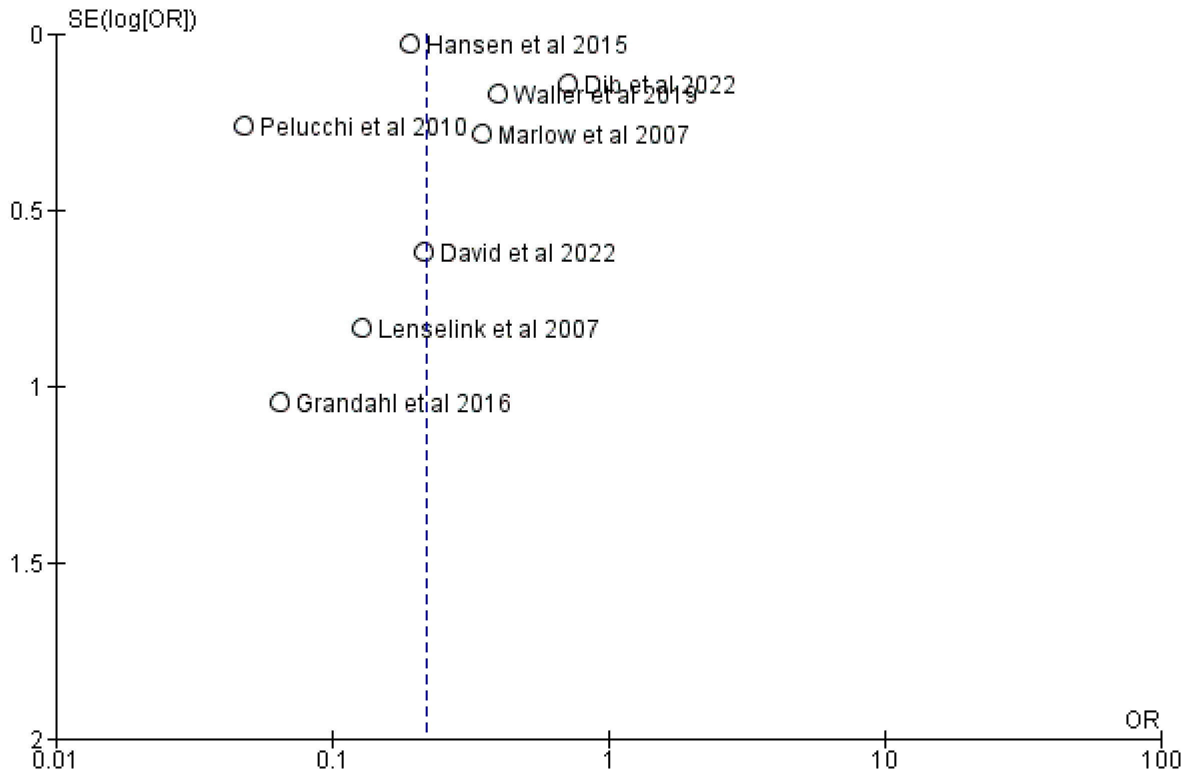

Figure S16: Funnel plot for the history of childhood vaccination comparison

Table S13: Sensitivity analysis for History of childhood vaccination for children

Study	Overall OR	Z	P	95% CI	I2	Initial CI
David et al 2022	0.22	4.64	< 0.00001	0.11 - 0.42	95%	0.12 - 0.40
Dib et al 2022	0.18	6.16	< 0.00001	0.10 - 0.31	89%	
Grandahl et al 2016	0.23	4.56	< 0.00001	0.23 - 0.44	95%	
Hansen et al 2015	0.21	3.62	0.0003	0.09 - 0.49	93%	
Lenselink et al 2007	0.23	4.59	< 0.00001	0.12 - 0.43	95%	
Marlow et al 2007	0.20	4.66	< 0.00001	0.10 - 0.39	95%	
Pelucchi et al 2010	0.29	3.97	< 0.0001	0.16 - 0.53	94%	
Waller et al 2019	0.19	4.51	< 0.00001	0.09 - 0.39	95%	

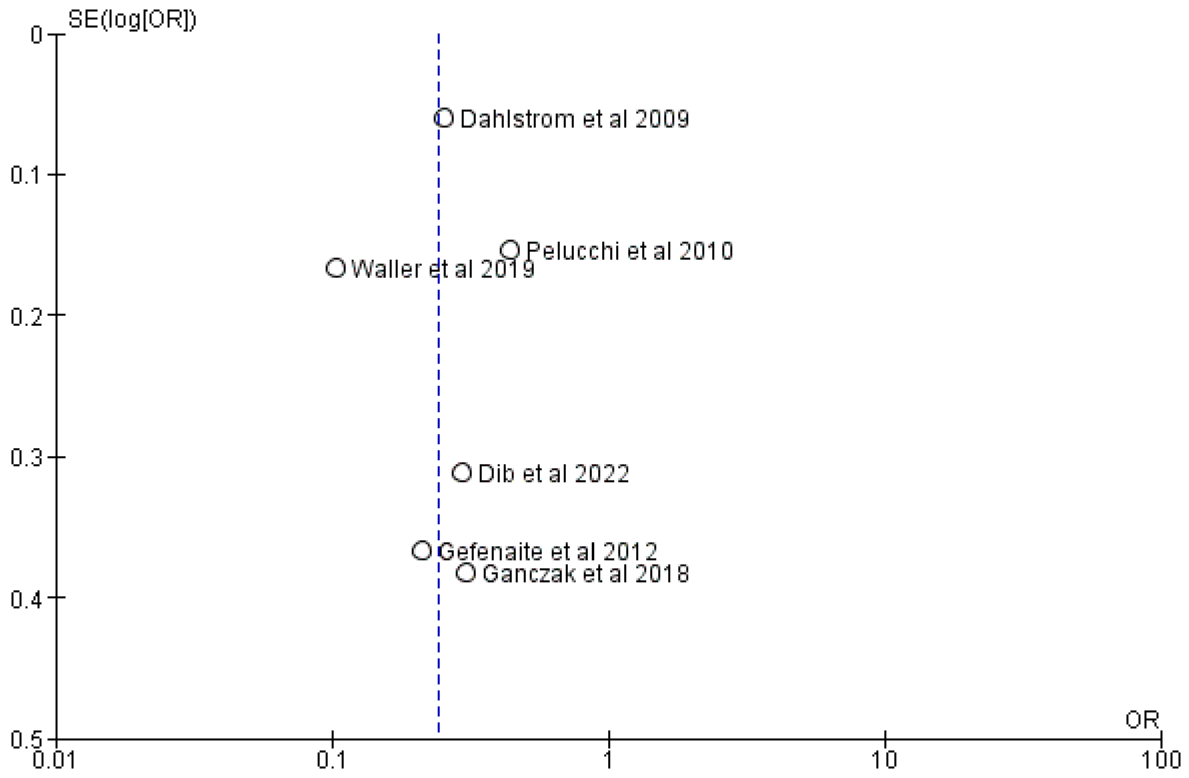

Figure S17: Funnel plot for the perceived efficacy comparison

Table S14: Sensitivity analysis for HPV vaccine efficacy comparison

Study	Overall OR	Z	P	95% CI	I2	Initial CI
Dahlstrom et al 2009	0.24	3.97	< 0.0001	0.12 - 0.49	91%	0.16 - 0.37
Dib et al 2022	0.24	5.79	< 0.00001	0.14 - 0.38	91%	
Ganczak et al 2018	0.24	5.94	< 0.00001	0.15 - 0.38	91%	
Gefenaite et al 2012	0.25	5.67	< 0.00001	0.15 - 0.40	91%	
Pelucchi et al 2010	0.21	6.39	< 0.00001	0.13 - 0.34	86%	
Waller et al 2019	0.30	7.60	< 0.00001	0.22 - 0.41	66%	

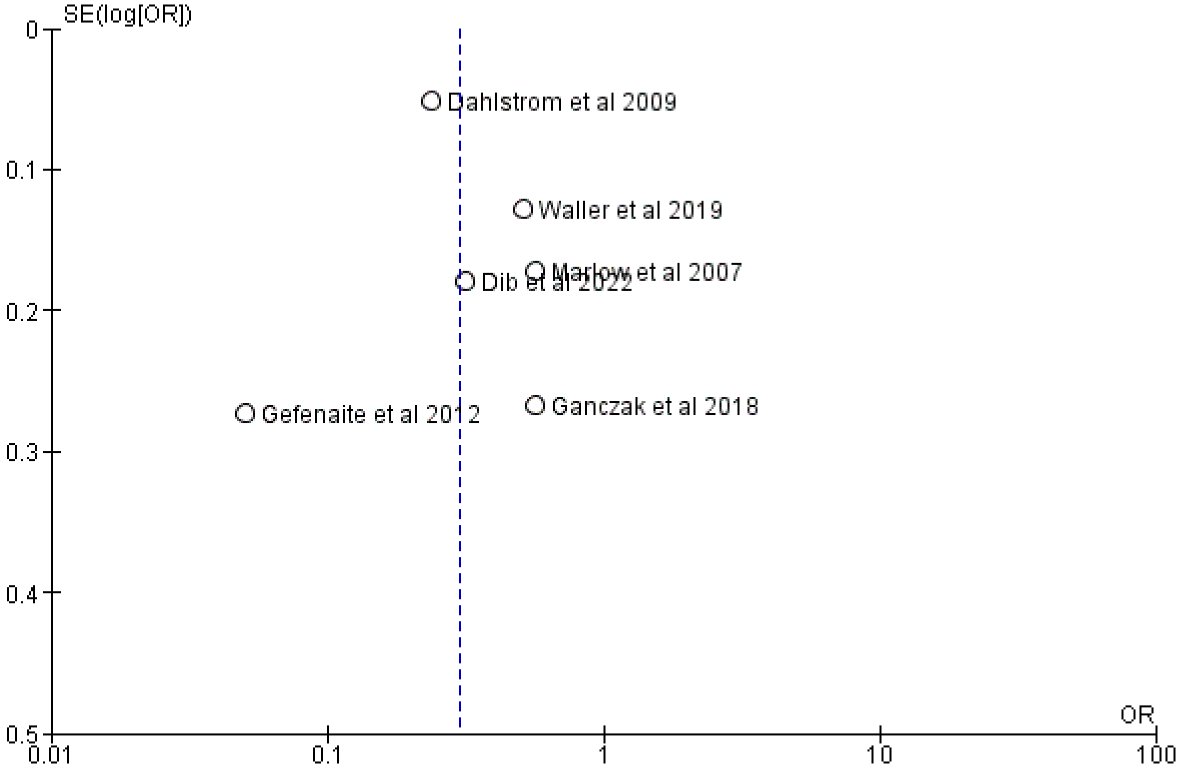

Figure S18: Funnel plot for the perceived safety comparison

Table S15: Sensitivity analysis for perceived safety of the HPV vaccine

Study	Overall OR	Z	P	95% CI	I2	Initial CI
Dahlstrom et al 2009	0.31	3.31	0.0009	0.16 - 0.62	94%	0.18 - 0.50
Dib et al 2022	0.30	3.94	< 0.0001	0.16 - 0.54	96%	
Ganczak et al 2018	0.27	4.69	< 0.00001	0.15 - 0.46	95%	
Gefenaite et al 2012	0.41	3.99	< 0.0001	0.26 - 0.63	93%	
Marlow et al 2007	0.26	4.73	< 0.00001	0.15 - 0.46	95%	
Waller et al 2019	0.27	4.45	< 0.00001	0.15 - 0.48	94%	

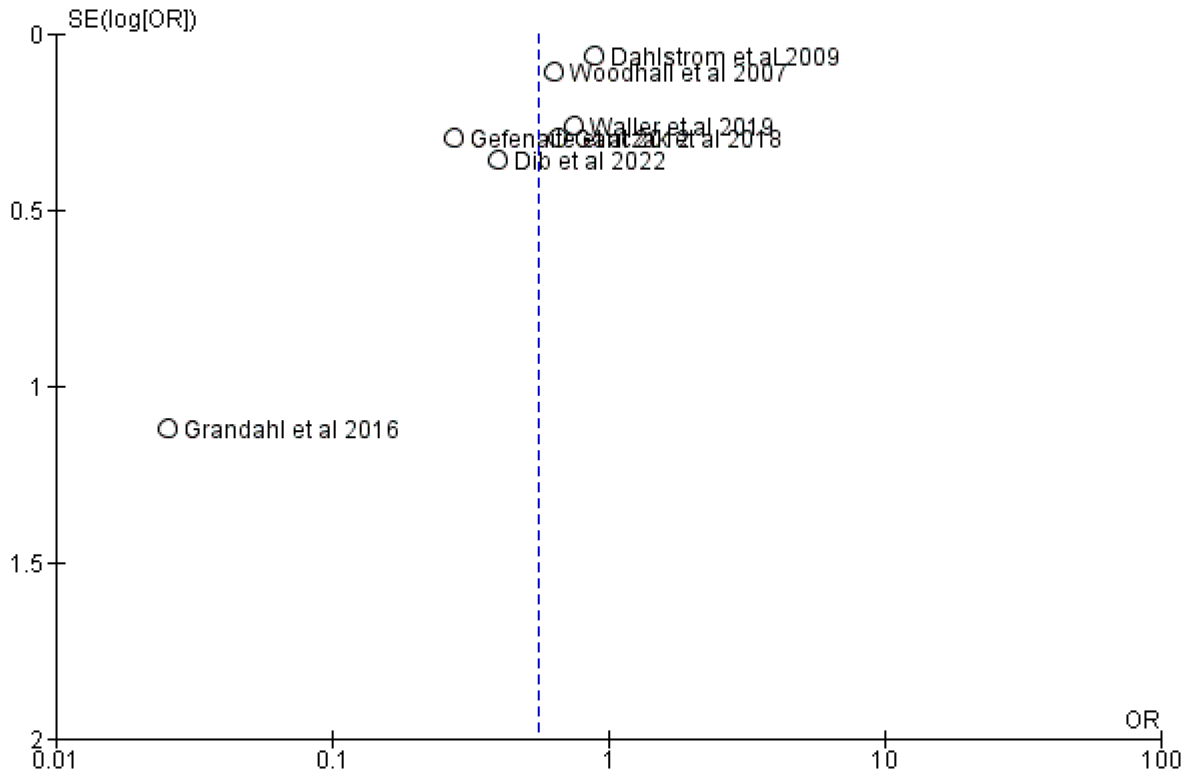

Figure S19: Funnel plot for the perceived increased risk behavior after vaccination comparison

Table S16: Sensitivity analysis for the perceived increased risk behavior after vaccination comparison

Study	Overall OR	Z	P	95% CI	I2	Initial CI
Dahlstrom et al 2009	0.48	3.44	0.0006	0.31 - 0.73	71%	0.39 - 0.79
Dib et al 2022	0.58	2.90	0.004	0.40 - 0.84	83%	
Ganczak et al 2018	0.53	3.14	0.002	0.36 - 0.79	85%	
Gefenaite et al 2012	0.64	2.71	0.007	0.47 - 0.89	76%	
Grandahl et al 2016	0.61	3.17	0.002	0.45 - 0.83	79%	
Waller et al 2019	0.52	3.22	0.001	0.35 - 0.77	85%	
Woodhall et al 2007	0.49	2.67	0.008	0.29 - 0.83	83%	

Study or Subgroup	Single/divorced/widowed Events	Total	Married/in a relationship Events	Total	Weight	Odds Ratio M-H, Random, 95% CI
**11.2.1 Mediocre quality (NOS: 5-7)**						
Baumann et al 2019	525	657	2465	2895	18.2%	0.69 [0.56, 0.86]
Dahlstrom et al 2009	2513	3195	7854	10389	21.4%	1.19 [1.08, 1.31]
Ganczak et al 2018	100	116	283	334	8.0%	1.13 [0.61, 2.06]
Grandahl et al 2016	47	51	140	150	2.7%	0.84 [0.25, 2.80]
Haesebaert et al 2014	14	18	41	73	2.8%	2.73 [0.82, 9.10]
Lenselink et al 2007	26	29	287	327	2.6%	1.21 [0.35, 4.17]
**Subtotal (95% CI)**		**4066**		**14168**	**55.7%**	**1.04 [0.72, 1.50]**
Total events	3225		11070			
Heterogeneity: Tau² = 0.10; Chi² = 22.37, df = 5 (P = 0.0004); I² = 78%						
Test for overall effect: Z = 0.22 (P = 0.83)						
**11.2.2 High quality (NOS: 8-10)**						
Hansen et al 2015	26426	33415	43791	56142	22.3%	1.07 [1.03, 1.10]
Schreiber et al 2015	17504	19107	43619	46573	22.0%	0.74 [0.69, 0.79]
**Subtotal (95% CI)**		**52522**		**102715**	**44.3%**	**0.89 [0.62, 1.27]**
Total events	43930		87410			
Heterogeneity: Tau² = 0.07; Chi² = 101.03, df = 1 (P < 0.00001); I² = 99%						
Test for overall effect: Z = 0.64 (P = 0.52)						
**Total (95% CI)**		**56588**		**116883**	**100.0%**	**0.96 [0.77, 1.18]**
Total events	47155		98480			
Heterogeneity: Tau² = 0.05; Chi² = 128.44, df = 7 (P < 0.00001); I² = 95%						
Test for overall effect: Z = 0.40 (P = 0.69)						
Test for subgroup differences: Chi² = 0.37, df = 1 (P = 0.54), I² = 0%						

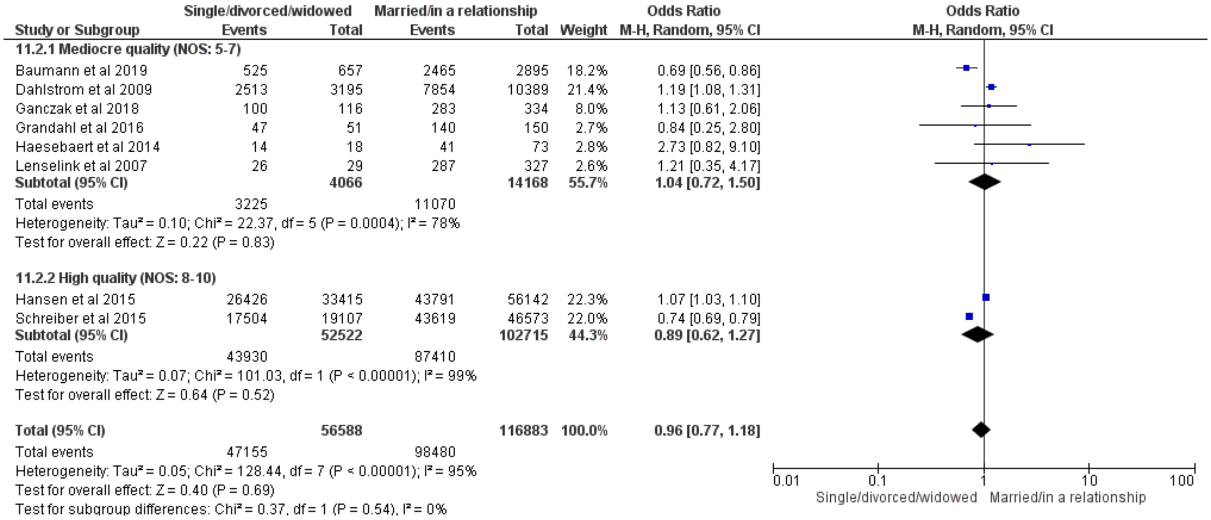

Figure S20: Forrest plot for the parents' civil status with subgroups based on the NOS scoring

Study or Subgroup	Unemployed parent Events	Total	Employed parent Events	Total	Weight	Odds Ratio M-H, Random, 95% CI
**7.2.1 Mediocre quality (NOS: 5-7)**						
Baumann et al 2019	322	632	2577	2921	22.8%	0.14 [0.11, 0.17]
Ganczak et al 2018	83	105	300	345	17.9%	0.57 [0.32, 1.00]
Haesebaert et al 2014	15	24	40	67	12.3%	1.13 [0.43, 2.94]
**Subtotal (95% CI)**		**761**		**3333**	**53.0%**	**0.42 [0.11, 1.54]**
Total events	420		2917			
Heterogeneity: Tau² = 1.21; Chi² = 36.90, df = 2 (P < 0.00001); I² = 95%						
Test for overall effect: Z = 1.31 (P = 0.19)						
**7.2.2 High quality (NOS: 8-10)**						
Hansen et al 2015	15507	21098	74296	93597	23.6%	0.72 [0.70, 0.75]
Schreiber et al 2015	7401	8397	52169	55489	23.5%	0.47 [0.44, 0.51]
**Subtotal (95% CI)**		**29495**		**149086**	**47.0%**	**0.58 [0.39, 0.88]**
Total events	22908		126465			
Heterogeneity: Tau² = 0.09; Chi² = 100.58, df = 1 (P < 0.00001); I² = 99%						
Test for overall effect: Z = 2.55 (P = 0.01)						
**Total (95% CI)**		**30256**		**152419**	**100.0%**	**0.45 [0.28, 0.74]**
Total events	23328		129382			
Heterogeneity: Tau² = 0.26; Chi² = 353.09, df = 4 (P < 0.00001); I² = 99%						
Test for overall effect: Z = 3.18 (P = 0.001)						
Test for subgroup differences: Chi² = 0.23, df = 1 (P = 0.63), I² = 0%						

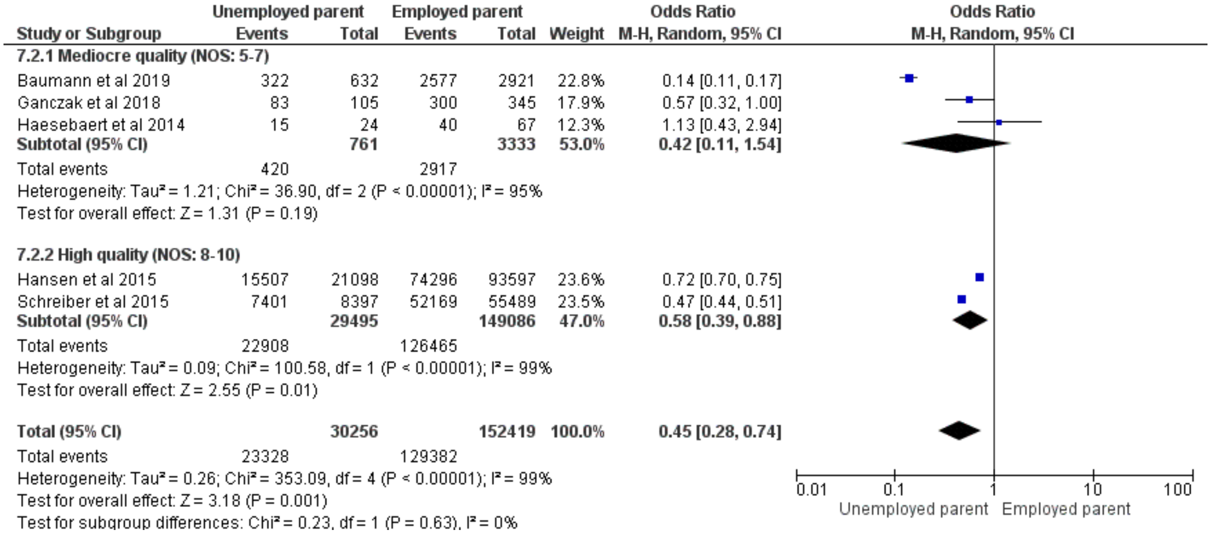

Figure S21: Forrest plot for the parents' employment status with subgroups based on the NOS scoring

Study or Subgroup	Immigrant Events	Immigrant Total	Native Events	Native Total	Weight	Odds Ratio M-H, Random, 95% CI
**5.2.1 Mediocre quality (NOS: 5-7)**						
Baumann et al 2019	130	168	2765	3384	16.7%	0.77 [0.53, 1.11]
Dahlstrom et al 2009	1192	1769	9175	11815	18.5%	0.59 [0.53, 0.66]
Grandahl et al 2016	16	18	170	182	6.0%	0.56 [0.12, 2.75]
Lenselink et al 2007	13	16	300	340	7.7%	0.58 [0.16, 2.12]
Mari et al 2022	32	50	219	289	13.9%	0.57 [0.30, 1.07]
**Subtotal (95% CI)**		**2021**		**16010**	**62.8%**	**0.60 [0.55, 0.67]**
Total events	1383		12629			
Heterogeneity: Tau² = 0.00; Chi² = 1.70, df = 4 (P = 0.79); I² = 0%						
Test for overall effect: Z = 9.64 (P < 0.00001)						
**5.2.2 High quality (NOS: 8-10)**						
Hansen et al 2015	10423	13430	59861	76256	18.6%	0.95 [0.91, 0.99]
Schreiber et al 2015	5505	6522	55657	59404	18.6%	0.36 [0.34, 0.39]
**Subtotal (95% CI)**		**19952**		**135660**	**37.2%**	**0.59 [0.23, 1.51]**
Total events	15928		115518			
Heterogeneity: Tau² = 0.46; Chi² = 473.79, df = 1 (P < 0.00001); I² = 100%						
Test for overall effect: Z = 1.10 (P = 0.27)						
**Total (95% CI)**		**21973**		**151670**	**100.0%**	**0.61 [0.38, 0.98]**
Total events	17311		128147			
Heterogeneity: Tau² = 0.31; Chi² = 488.33, df = 6 (P < 0.00001); I² = 99%						
Test for overall effect: Z = 2.05 (P = 0.04)						
Test for subgroup differences: Chi² = 0.00, df = 1 (P = 0.95), I² = 0%						

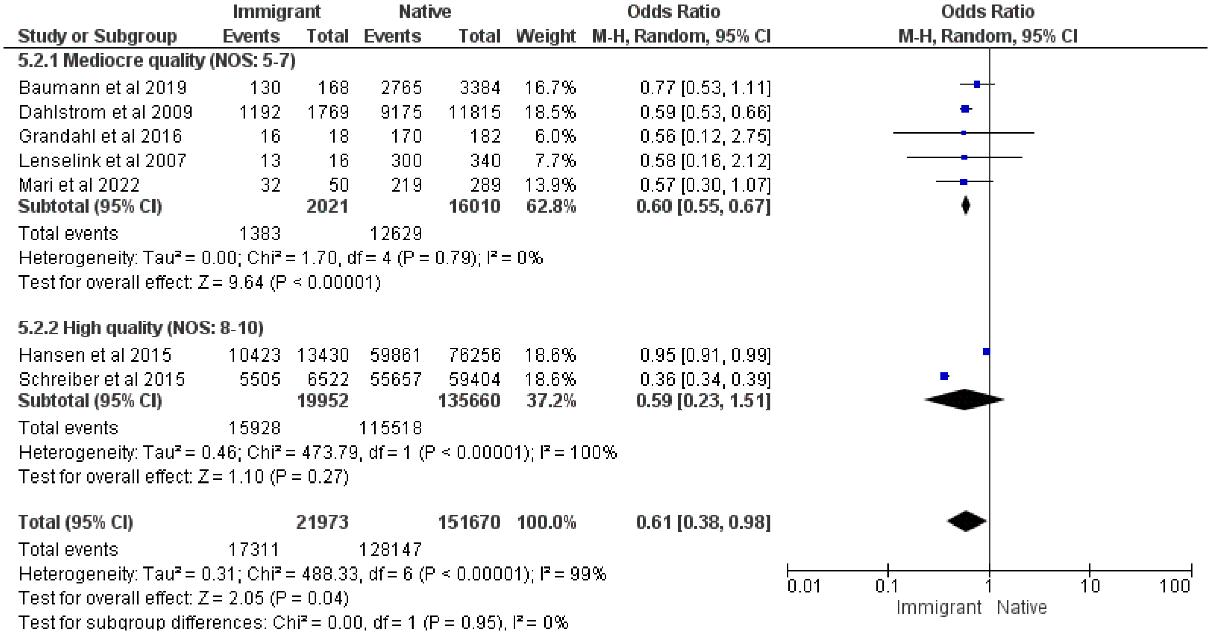

Figure S22: Forrest plot for the parents' immigrant status with subgroups based on the NOS scoring

Study or Subgroup	Mom does not attend CCS Events	Mom does not attend CCS Total	Mom attends CCS Events	Mom attends CCS Total	Weight	Odds Ratio M-H, Random, 95% CI
**14.2.1 Mediocre quality (NOS: 5-7)**						
David et al 2022	6	12	17	45	10.8%	1.65 [0.46, 5.94]
Haesebaert et al 2014	11	16	44	75	11.9%	1.55 [0.49, 4.91]
Lenselink et al 2007	13	14	300	342	6.2%	1.82 [0.23, 14.27]
Rousset-Jablonski et al 2021	10	42	24	68	14.6%	0.57 [0.24, 1.36]
**Subtotal (95% CI)**		**84**		**530**	**43.4%**	**1.02 [0.56, 1.83]**
Total events	40		385			
Heterogeneity: Tau² = 0.01; Chi² = 3.05, df = 3 (P = 0.38); I² = 2%						
Test for overall effect: Z = 0.05 (P = 0.96)						
**14.2.2 High quality (NOS: 8-10)**						
Gefenaite et al 2012	23	33	283	435	15.5%	1.24 [0.57, 2.66]
Hansen et al 2015	13884	18390	56996	72150	20.6%	0.82 [0.79, 0.85]
Spencer et al 2007	1053	1935	88035	110516	20.5%	0.30 [0.28, 0.33]
**Subtotal (95% CI)**		**20358**		**183101**	**56.6%**	**0.64 [0.28, 1.46]**
Total events	14960		145314			
Heterogeneity: Tau² = 0.48; Chi² = 391.17, df = 2 (P < 0.00001); I² = 99%						
Test for overall effect: Z = 1.06 (P = 0.29)						
**Total (95% CI)**		**20442**		**183631**	**100.0%**	**0.83 [0.45, 1.52]**
Total events	15000		145699			
Heterogeneity: Tau² = 0.47; Chi² = 395.67, df = 6 (P < 0.00001); I² = 98%						
Test for overall effect: Z = 0.61 (P = 0.54)						
Test for subgroup differences: Chi² = 0.79, df = 1 (P = 0.37), I² = 0%						

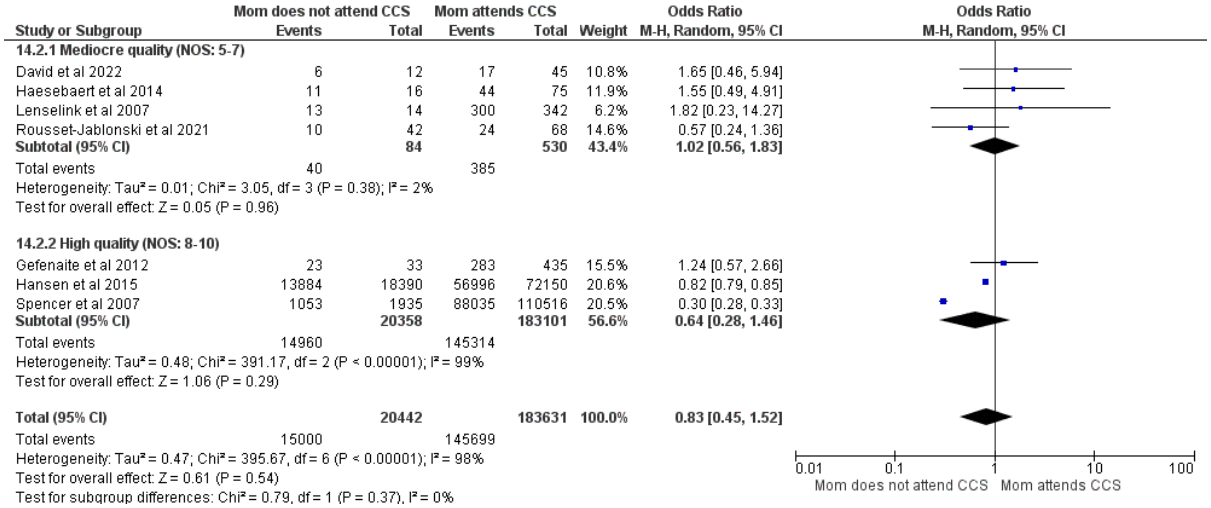

Figure S23: Forrest plot for the mother's previous CCS attendance with subgroups based on the NOS scoring

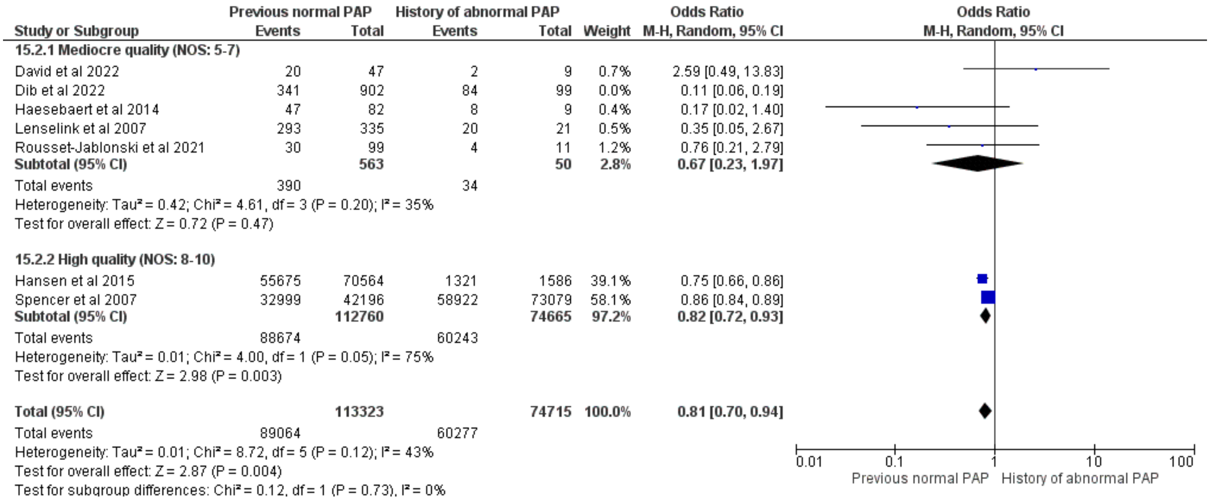

Figure S24: Forrest plot for the mother's previous PAP results with subgroups based on the NOS scoring

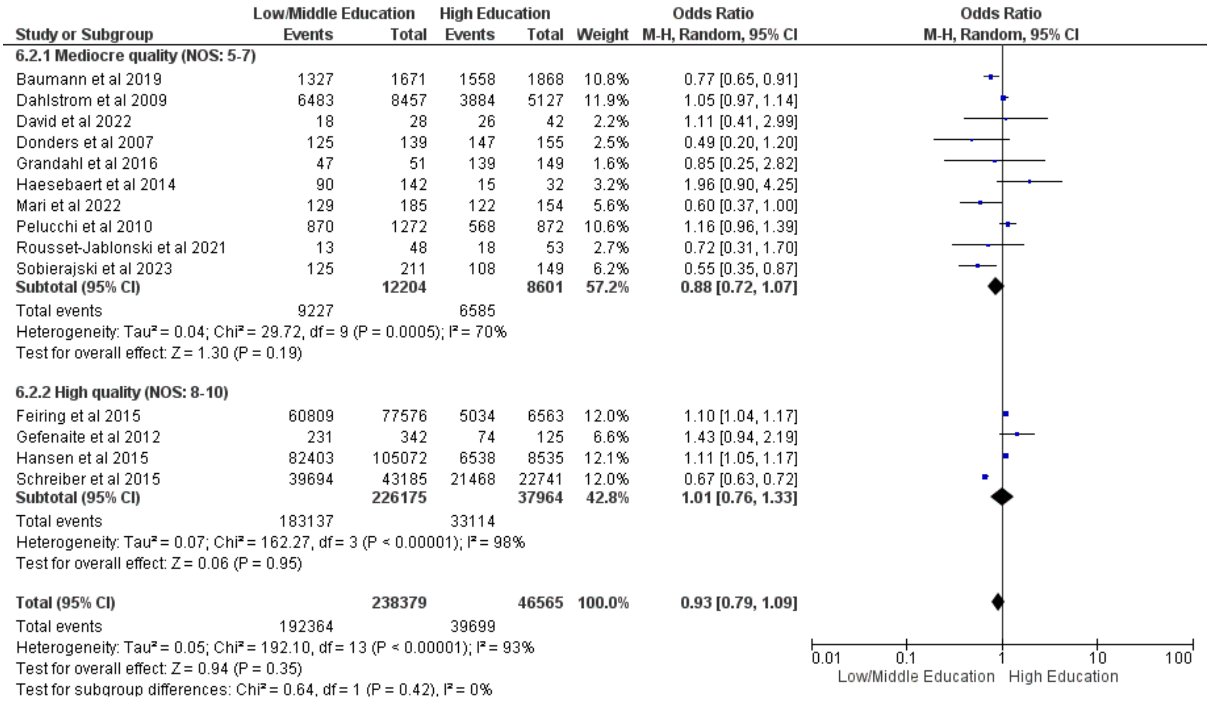

Figure S25: Forrest plot for the parents' education levels with subgroups based on the NOS scoring
